# Generation and characterization of β1,2-gluco-oligosaccharide probes from *Brucella abortus* cyclic β-glucan and their recognition by C-type lectins of the immune system

**DOI:** 10.1093/glycob/cww041

**Published:** 2016-10-18

**Authors:** Hongtao Zhang, Angelina S Palma, Yibing Zhang, Robert A Childs, Yan Liu, Daniel A Mitchell, Leticia S Guidolin, Wilfried Weigel, Barbara Mulloy, Andrés E Ciocchini, Ten Feizi, Wengang Chai

**Affiliations:** 2 Glycosciences Laboratory, Department of Medicine, Imperial College London, London W12 0NN, UK; 3 Key Laboratory of Carbohydrate Chemistry and Biotechnology, Ministry of Education, School of Biotechnology, Jiangnan University, Wuxi 214122, China; 4 UCIBIO-REQUIMTE, Department of Chemistry, Faculty of Science and Technology, NOVA Universidade de Lisboa, Caparica 2829-516, Portugal; 5 CSRI-UHCW, Walsgrave Campus, University of Warwick, Coventry CV2 2DX, UK; 6 Instituto de Investigaciones Biotecnológicas “Dr. Rodolfo A. Ugalde”, Instituto Tecnológico de Chascomús (IIB-INTECH), Universidad Nacional de San Martín, San Martín, Buenos Aires 1650, Argentina; 7 SCIENION AG, Volmerstrasse 7b, Berlin 12489, Germany

**Keywords:** β1,2-glucan, carbohydrate microarray, C-type lectins, glucan recognition, neoglycolipids

## Abstract

The β1,2-glucans produced by bacteria are important in invasion, survival and immunomodulation in infected hosts be they mammals or plants. However, there has been a lack of information on proteins which recognize these molecules. This is partly due to the extremely limited availability of the sequence-defined oligosaccharides and derived probes for use in the study of their interactions. Here we have used the cyclic β1,2-glucan (CβG) of the bacterial pathogen *Brucella abortus*, after removal of succinyl side chains, to prepare linearized oligosaccharides which were used to generate microarrays. We describe optimized conditions for partial depolymerization of the cyclic glucan by acid hydrolysis and conversion of the β1,2-gluco-oligosaccharides, with degrees of polymerization 2–13, to neoglycolipids for the purpose of generating microarrays. By microarray analyses, we show that the C-type lectin receptor DC-SIGNR, like the closely related DC-SIGN we investigated earlier, binds to the β1,2-gluco-oligosaccharides, as does the soluble immune effector serum mannose-binding protein. Exploratory studies with DC-SIGN are suggestive of the recognition also of the intact CβG by this receptor. These findings open the way to unravelling mechanisms of immunomodulation mediated by β1,2-glucans in mammalian systems.

## Introduction

Glucan polysaccharides are of biomedical interest because of their involvement in mechanisms of pathogen recognition and modulation of the immune system (Brown and Gordon 2003; Chen and Seviour 2007). Molecular dissection of their interactions with proteins of the immune system, although desirable is not straightforward at the level of polysaccharides on account of the inherent heterogeneities of these macromolecules. With the advent of oligosaccharide microarray technologies (Fukui et al. 2002; Blixt et al. 2004; Feizi and Chai 2004; Rillahan and Paulson 2011; Palma et al. 2014), it is possible now to explore interactions with proteins using oligosaccharide probes generated from a range of oligosaccharide sequences that can be prepared after partial depolymerization of the polysaccharides (Pedersen et al. 2012; Palma et al. 2015).

The microarray system based on the neoglycolipid (NGL) technology (Chai et al. 2003) for preparing lipid-linked oligosaccharide probes for immobilization and binding studies, lends itself well to analyses of glucan sequences as recognition structures within polysaccharides. This is the basis of the “designer” microarray approach (Palma et al. 2006, 2014; Gao et al. 2014) whereby microarrays are generated from oligosaccharides released from the targeted macromolecules; oligosaccharides bound by recognition proteins may be isolated for characterization. This approach was used successfully in studies of the ligands on glucan polysaccharides for Dectin-1, a key receptor of the innate immune system directed against fungal pathogens (Herre et al. 2004). Dectin-1 belongs to the family of C (calcium-dependent)-type *lectin-like* proteins; it lacks the canonical amino acid residues for ligating calcium, required for carbohydrate binding in classical C-type lectins (Drickamer and Taylor, 2015). Nevertheless, designer microarrays (Palma et al. 2006) generated from oligosaccharide fractions derived from fungal-type glucans (Brown and Gordon, 2001; Brown et al. 2003), established that (i) dectin-1 is a calcium-independent carbohydrate-binding protein and (ii) linear β1,3-linked glucose sequences with degrees of polymerization (DP) 10 or longer are required for detection of binding.

Using the designer approach, in conjunction with a novel high-sensitivity mass spectrometric (MS) sequencing method, we recently generated a “glucome” microarray of sequence-defined oligosaccharide probes derived from glucan polysaccharides of fungal, bacterial and plant origins in order to use as a high-throughput screening tool for characterizing glucan recognition systems of mammals and bacteria (Palma et al. 2015). The probes in the microarray encompassed linear sequences with a single linkage type: 1,2-, 1,3-, 1,4- or 1,6- with α or β configurations; and mixed multiple linkage types: 1,3-, 1,4 or 1,6-; also branched oligosaccharide sequences with 1,3 and 1,6-linkages with different DPs. Binding of the dendritic cell-specific C-type lectin receptor (CLR) DC-SIGN was noted to NGL probes from β1,2-linked gluco-oligosaccharides DP 2–13, derived from the cyclic β1,2-glucan (CβG) of the bacterial pathogen *Brucella abortus*, which is a major pathogenic factor involved in *B. abortus* invasion and survival (Arellano-Reynoso et al. 2005) and a potent activator of mouse and human dendritic cells (Martirosyan et al. 2012). This raised the possibility that DC-SIGN interacts with *B. abortu*s CβG and that this interaction participates in modulation of the activities of DCs (Palma et al. 2015). CLRs comprise a large family of signaling receptors, which are variously involved in inflammatory and innate immune responses to a diverse range of microbial pathogens (Hoving et al. 2014; Drickamer and Taylor 2015). These activities occur following the binding of their carbohydrate recognition domains (CRDs) to specific endogenous carbohydrates and those of pathogens. The finding that DC-SIGN can bind pathogen-associated β1,2-linked gluco-oligosaccharides raises the question whether related CLRs bind to these types of sequences, in addition to their other well-known carbohydrate ligands.

Here we describe details of the preparation of sequence-defined β1,2-linked gluco-oligosaccharide probes for microarray analysis, including procedures for CβG hydrolysis, oligosaccharide fractionation, with improved yields of NGLs from the longer oligosaccharides that are difficult to derivatize. We apply the NGL microarrays to investigate the recognition of these oligosaccharide sequences by C-type lectin immune-receptors, including DC-SIGN and its closely related human receptor DC-SIGNR (or L-SIGN), and the soluble serum effector mannose-binding protein (MBP). We also explore the recognition of the intact cyclic forms of CβGs by DC-SIGN.

## Results

### Preparation of β1,2-gluco-oligosaccharides from cyclic β1,2-glucan

**Fig. 1.**
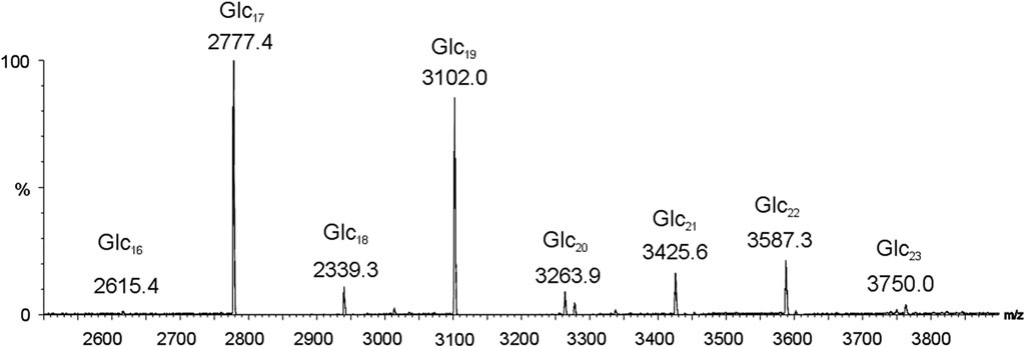
MALDI mass spectrum of CβG extracted from *Brucella abortus* after removal of the succinyl side chains by mild alkaline treatment.

The alkali treated *B. abortus* CβG was analyzed by MALDI-MS, and the spectrum indicated complete removal of the succinyl side chains and preservation of the cyclic glucan chains which consisted of DP 16–23, with DP 17 (MNa^+^ at *m*/*z* 2777) being the most abundant component (Figure 1).

**Fig. 2.**
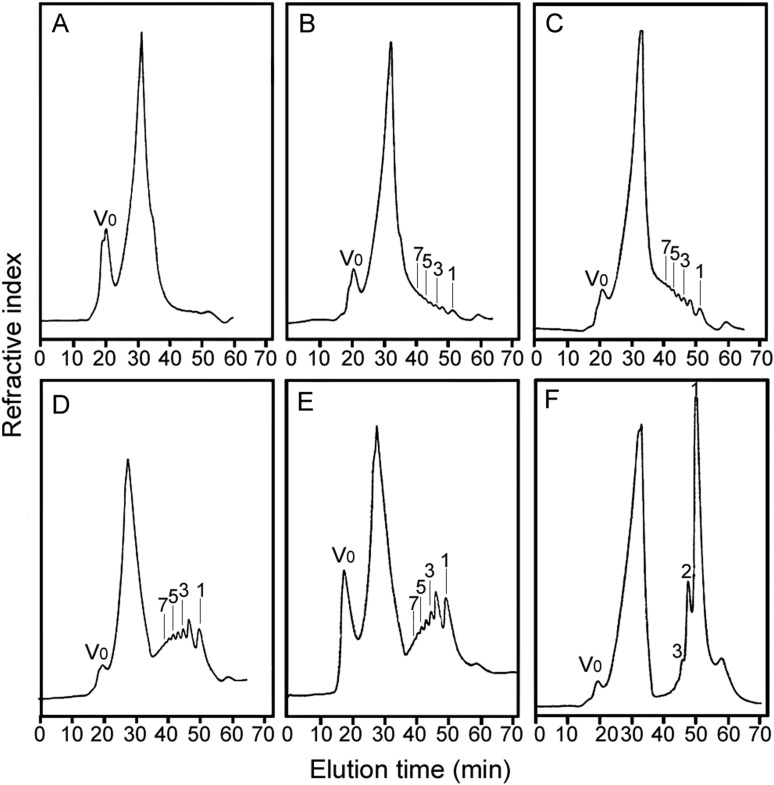
Analysis of hydrolysis products of CβG at different reaction time by gel filtration chromatography. (**A**) 0 min, (**B**) 30 min, (**C**) 60 min, (**D**) 120 min, (**E**) 180 min and (**F**) 210 min. Acid hydrolysis was carried out with 0.01 M HCl at 100°C in a V-shaped glass vial with stirring. For gel filtration, a Superdex Peptide column was used; the column was eluted with deionized water and the eluent was monitored by refractive index. The major peak at ∼30 min was an artifact, resulting from HCl present in the reaction mixture.

**Fig. 3.**
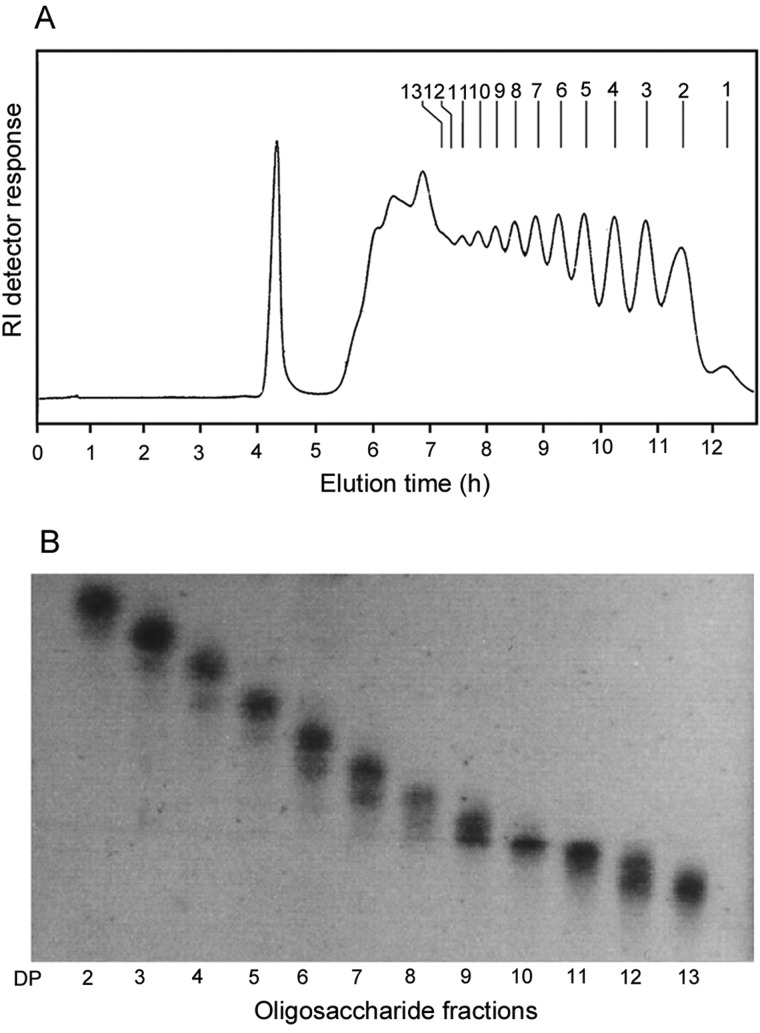
Preparation of CβG oligosaccharide fragments. (**A**) Bio-Gel P4 profile of CβG hydrolysate and (**B**) HPTLC analysis of aliquots from each collected fractions.

**Fig. 4.**
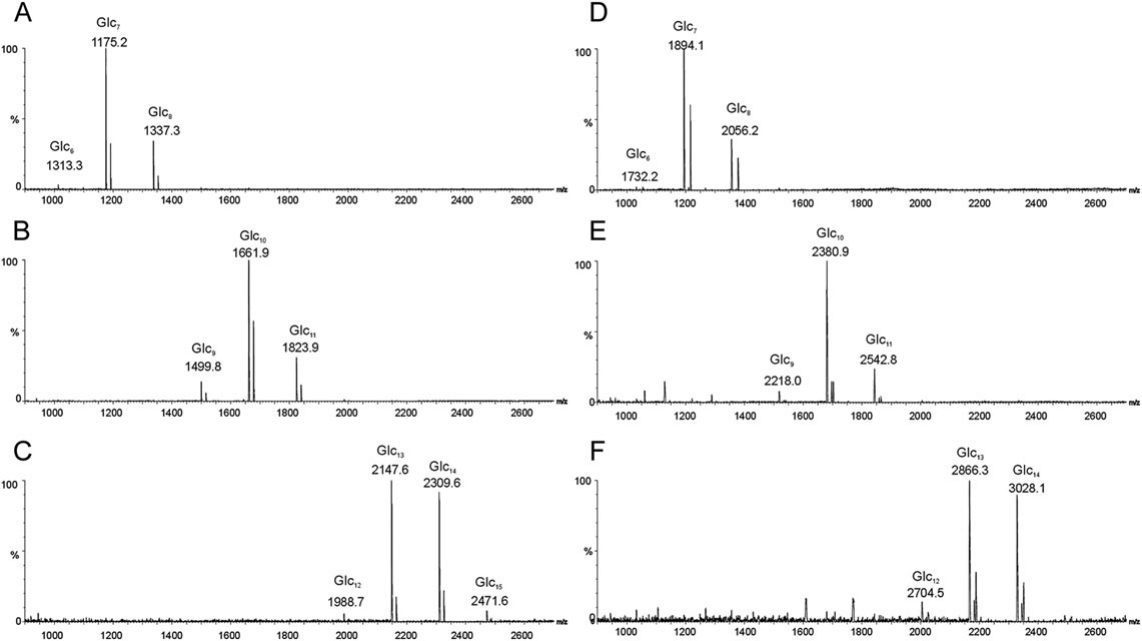
MALDI mass spectra of selected CβG oligosaccharides and their NGLs. (**A**) Heptasaccharide, (**B**) decasaccharide, (**C**) tridecasaccharide, (**D**) NGL of heptasaccharide, (**E**) NGL of decasaccharide and (**F**) NGL of tridecasaccharide.

In the exploratory small-scale experiments, hydrolysis of the CβG with 0.01 M HCl at 100°C was assessed by monitoring the products at different reaction times by gel filtration (Figure 2). For monitoring of the reaction, the reagent HCl was not removed prior to analysis, and therefore an artefactual peak related to HCl occurred at ∼30 min. This has not interfered with the evaluation of the progress of the hydrolysis. The reaction time of 120 min (Figure 2D) was selected for large-scale experiments to obtain oligosaccharides with DPs ranging from 2 to 13 (Figure 3A). The fractions obtained by gel filtration were analyzed by HPTLC (Figure 3B). The identities of the major components in the higher oligosaccharide fractions with DPs ≥ 5 were determined by MALDI-MS and of the lower oligosaccharide fractions with DPs ≤ 4 by negative-ion ESI-MS. As shown in the MALDI spectra of fractions DP 7, 10 and 13 (Figure 4A–C, respectively) as representative, each fraction contains adjacent overlapping components in addition to the main component. For example, in fraction DP 7 (Figure 4A), oligosaccharides with DP 6 and 8 were present as minor components in addition to the main component DP 7 at *m*/*z* 1175.2 (MNa^+^), due to incomplete separation by gel filtration chromatography.

**Fig. 5.**
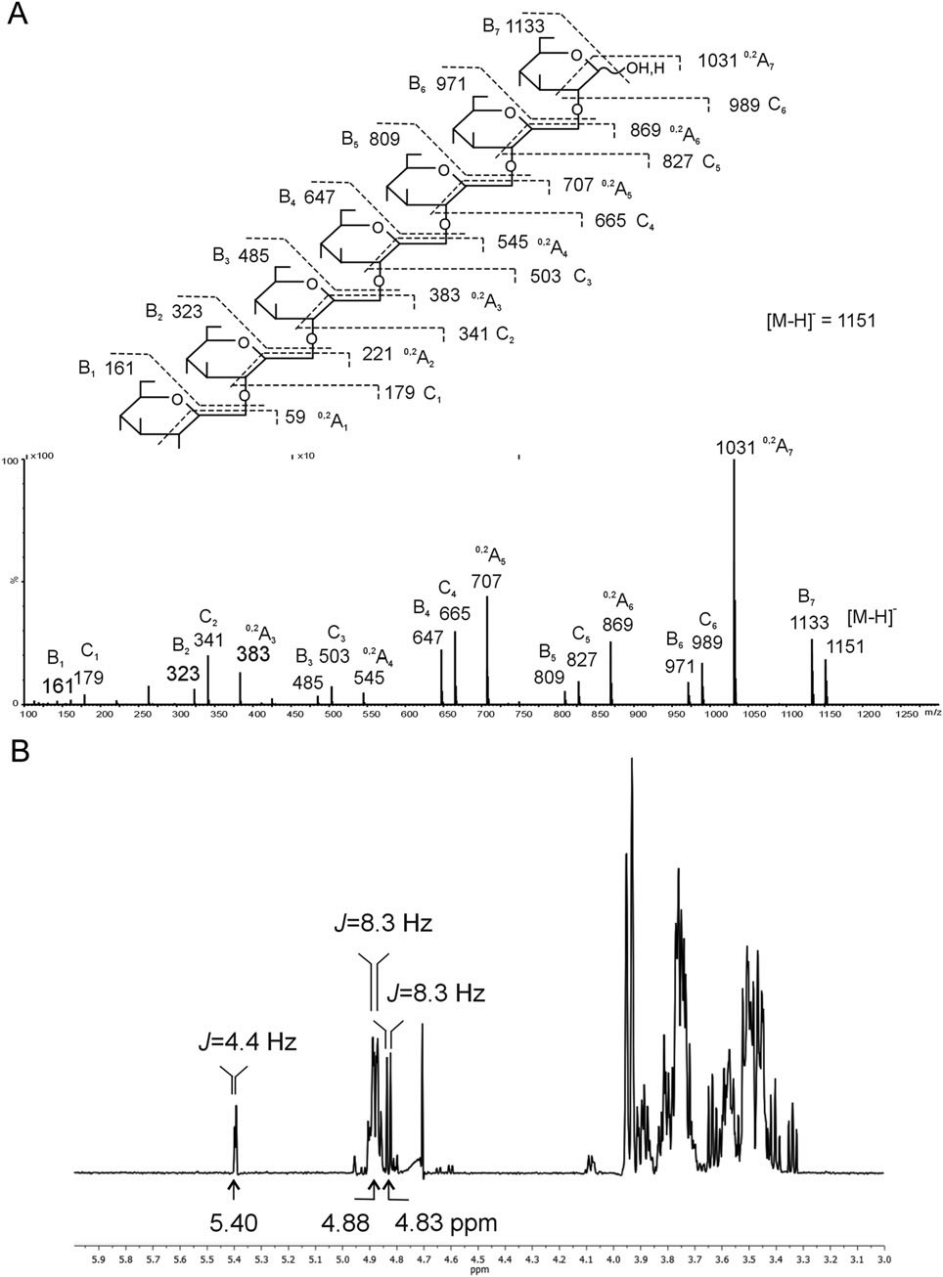
Sequence analysis of CβG heptasaccharide by negative-ion ESI-CID-MS/MS (**A**) and ^1^H NMR (**B**). The heptasaccharide structure is shown to indicate fragmentation (A). The major doublet at 4.88 ppm with a coupling constant of 8.3 Hz was used to assign the β-anomeric configuration; anomeric signals arising from the reducing end monosaccharide were also identified as follows: *α*: 5.40 ppm, 4.4 Hz; *β*: 4.83 ppm, 8.3 Hz (B).

Linkage and anomeric configuration for the DP 7 fraction were investigated by negative-ion ESI-CID-MS/MS and ^1^H NMR. In the product-ion spectrum (Figure 5A), the neutral losses of 18 Da (e.g. *m*/*z* 1133 and 971) and 120 Da (e.g. *m*/*z* 1031 and 869) derived from dehydration and ^0,2^A-cleavage (Domon and Costello 1988), respectively, of the [M − H]^−^ and glycosidic C-type ions (Domon and Costello 1988) are characteristic of 1,2-linkage of gluco-oligosaccharides (Palma et al. 2015). The β-anomeric configuration could be readily assigned by ^1^H NMR from the major anomeric doublet at 4.88 ppm with a coupling constant of ∼8.3 Hz; both α- and β-anomeric signals from the reducing end monosaccharide could also be identified (Figure 5B).

### Preparation of β1,2-gluco-oligosaccharide NGLs

Preparation of the NGLs of glucan oligosaccharides with DP > 7 using the conventional method of reductive-amination (Chai et al. 2003) has been difficult and the yield extremely low (not shown). For the higher oligomers of gluco-oligosaccharides even with the relatively more efficient reaction in oxime-ligation (Liu et al. 2007) the yield was again low. Improvement of conjugation conditions was attempted by modifications of several parameters of the oxime-ligation reaction. Using the readily available α1,6-linked dextran oligosaccharides as standards, we explored the effects of different reaction temperature (22, 50 and 80°C) and time (24, 48 and 96 h), different acidity of the reaction medium (acidic, neutral and alkaline) and different amounts of lipid reagent, but no major improvement in reaction yield was found (not shown).

**Fig. 6.**
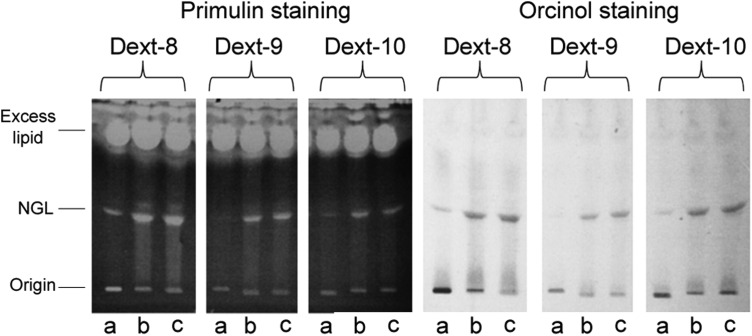
Optimization of conjugation reaction conditions. (a) The reaction condition (a) was used, (b) the reaction condition (b) was used and (c) the reaction condition (c) was used. Details of conditions (a), (b) and (c) are described in *Materials and methods*. Lipid was revealed by fluorescence of primulin staining and hexose by orcinol staining.

The low solubility of gluco-oligosaccharides being a well-recognized problem, we next investigated the effect of solvent on conjugation yield. To improve the solubility of gluco-oligosaccharides, DMSO was included in the solvent mixture for NGL conjugation. Using dextran oligosaccharides with DP 8, 9 and 10 as examples, the solvent effect was clearly apparent. In the presence of DMSO, the yields were improved, particularly for the higher oligomers. As shown in Figure 6, the NGL product bands in lanes b and c, in which DMSO was included in the reaction solvent, were clearly more intense than those in lanes a. This was apparent with both primulin (for detection of lipid) and orcinol (for detection of glucose) staining.

**Fig. 7.**
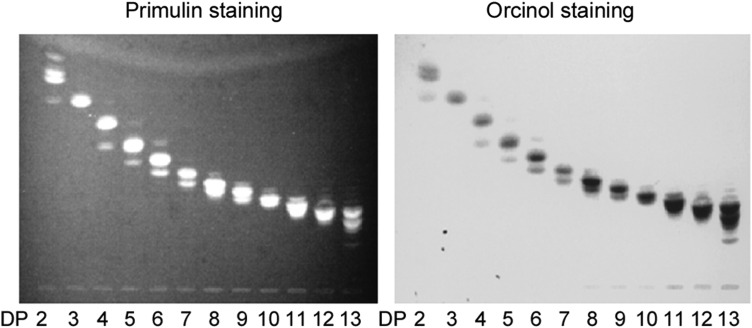
HPTLC analysis of CβG oligosaccharide NGLs. Two to 13 represent the DP of β1,2-gluco-oligosaccharides isolated from CβG.

With the modified conditions (condition c in *Materials and methods*), a series of NGLs of the CβG oligosaccharides, DP 2–13 was prepared. The purified NGL probes were analyzed by HPTLC (Figure 7) and MALDI-MS (Figure 4D–F) before printing on nitrocellulose-coated glass slides for protein-binding experiments.

### Analysis of the recognition of β1,2-gluco-oligosaccharide NGLs by CLRs of the immune system

**Fig. 8.**
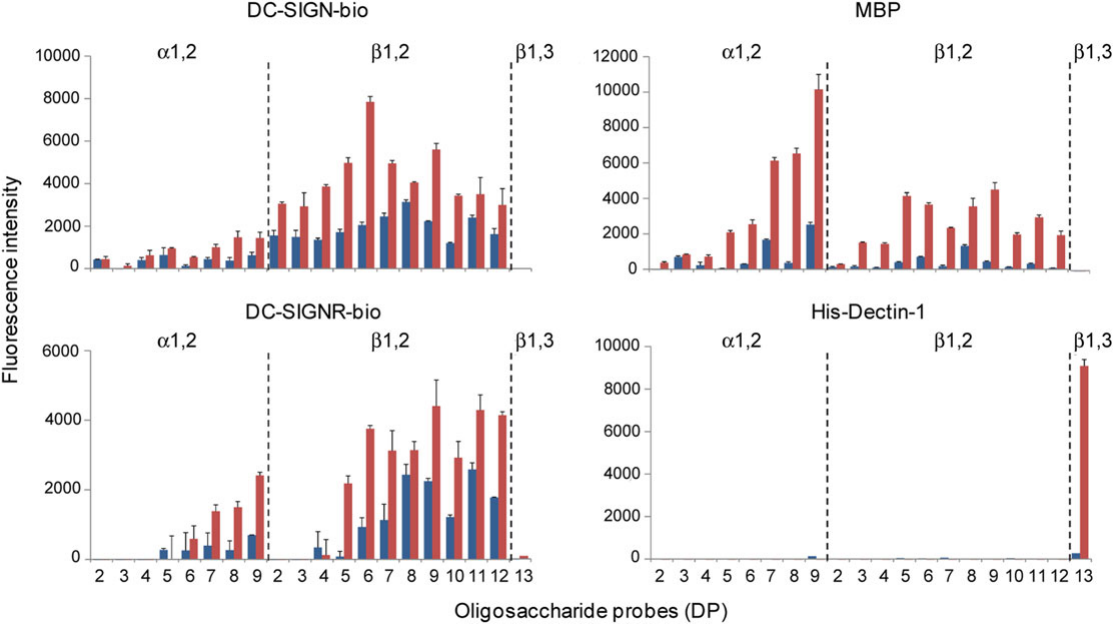
Carbohydrate microarray analysis of the interaction of C-type lectin receptors with CβG oligosaccharides. DC-SIGN-bio and DC-SIGNR-bio were tested at 50 µg mL^−1^, serum MBP at 4 µg mL^−1^ and His-Dectin-1 at 20 µg/ml. The microarray consisted of lipid-linked gluco-oligosaccharide probes (AO-NGLs) printed in duplicate on nitrocellulose-coated glass slides. The linkage type and DP of the major component are indicated; their sequences are shown in Table I. The results are the means of fluorescence intensities of duplicate spots, printed at 2 and 5 fmol spot^−1^ (black and dark grey, respectively), and with the range indicated by error bars. This figure is available in black and white in print and in color at *Glycobiology* online.

In order to investigate the recognition of the β1,2-gluco-oligosaccharides by CLRs of the immune system, we arrayed the gluco-oligosaccharides as NGLs and included α1,2-linked DP 2–9 and β1,3-linked DP 13 NGLs as controls (Table I). We performed microarray analyses with proteins: DC-SIGN-bio, DC-SIGNR-bio, MBP purified from human serum and His-Dectin-1 (Figure 8). DC-SIGN-bio showed binding to all the β1,2-gluco-oligosaccharide probes tested; the binding pattern was similar to that previously observed using DC-SIGN-Fc (Palma et al. 2015), namely with DP 6 showing the highest binding signal at 5 fmol glucan probe per spot (Supplementary, Figure S1). DC-SIGNR-bio gave binding signals with β1,2-gluco-oligosaccharide probes with DP > 4. MBP also showed binding to β1,2-gluco-oligosaccharide probes with DP > 2 with relatively high intensity to those with longer chain lengths, DP 7–9. Contrasting with binding profiles of DC-SIGN-bio and DC-SIGNR-bio, MBP showed relatively higher binding signals with the α1,2-gluco-oligosaccharide probes, in particular the longer oligomers. His-Dectin-1 included as a control in these microarray analyses showed the predicted binding to the β1,3-linked DP 13 from curdlan (Palma et al. 2006), but no detectable binding to any of the CβG derived β1,2-linked gluco-oligosaccharide probes (Figure 8), in agreement with our previous assignment (Palma et al. 2015). Under the assay conditions, DC-SIGN-bio, DC-SIGNR-bio and MBP gave no binding signals with the β1,3-linked DP 13 probe.

**Table I.** Gluco-oligosaccharide NGL probes included in the microarray

Name^a^	Sequence^b^	DP^c^
Cyano-2	Glcα1–2Glc-AO	2
Cyano-3	Glcα1–2Glcα1–2Glc-AO	3
Cyano-4	Glcα1–2Glcα1–2Glcα1–2Glc-AO	4
Cyano-5	Glcα1–2Glcα1–2Glcα1–2Glcα1–2Glc-AO	5
Cyano-6	Glcα1–2Glcα1–2Glcα1–2Glcα1–2Glcα1–2Glc-AO	6
Cyano-7	Glcα1–2Glcα1–2Glcα1–2Glcα1–2Glcα1–2Glcα1–2Glc-AO	7
Cyano-8	Glcα1–2Glcα1–2Glcα1–2Glcα1–2Glcα1–2Glcα1–2Glcα1–2Glc-AO	8
Cyano-9	Glcα1–2Glcα1–2Glcα1–2Glcα1–2Glcα1–2Glcα1–2Glcα1–2Glcα1–2Glc-AO	9
CβG-2	Glcβ1–2Glc-AO	2
CβG-3	Glcβ1–2Glcβ1–2Glc-AO	3
CβG-4	Glcβ1–2Glcβ1–2Glcβ1–2Glc-AO	4
CβG-5	Glcβ1–2Glcβ1–2Glcβ1–2Glcβ1–2Glc-AO	5
CβG-6	Glcβ1–2Glcβ1–2Glcβ1–2Glcβ1–2Glcβ1–2Glc-AO	6
CβG-7	Glcβ1–2Glcβ1–2Glcβ1–2Glcβ1–2Glcβ1–2Glcβ1–2Glc-AO	7
CβG-8	Glcβ1–2Glcβ1–2Glcβ1–2Glcβ1–2Glcβ1–2Glcβ1–2Glcβ1–2Glc-AO	8
CβG-9	Glcβ1–2Glcβ1–2Glcβ1–2Glcβ1–2Glcβ1–2Glcβ1–2Glcβ1–2Glcβ1–2Glc-AO	9
CβG-10	Glcβ1–2Glcβ1–2Glcβ1–2Glcβ1–2Glcβ1–2Glcβ1–2Glcβ1–2Glcβ1–2Glcβ1–2Glc-AO	10
CβG-11	Glcβ1–2Glcβ1–2Glcβ1–2Glcβ1–2Glcβ1–2Glcβ1–2Glcβ1–2Glcβ1–2Glcβ1–2Glcβ1–2Glc-AO	11
CβG-12	Glcβ1–2Glcβ1–2Glcβ1–2Glcβ1–2Glcβ1–2Glcβ1–2Glcβ1–2Glcβ1–2Glcβ1–2Glcβ1–2Glcβ1–2Glc-AO	12
Curd-13	Glcβ1-3Glcβ1-3Glcβ1-3Glcβ1-3Glcβ1-3Glcβ1-3Glcβ1-3Glcβ1-3Glcβ1-3Glcβ1-3Glcβ1-3Glcβ1-3Glc-AO	13

^a^Cyano: from cyanobacterium gluco-fructosides; CβG: from cyclic β-glucan of bacterium *Brucella abortus*; Curd: from curdlan polysaccharide.

^b^CβG-2 to CβG-7 and Curd-13: relatively pure (purity >90%, based on HPTLC and MALDI-MS analysis); CβG-8 to CβG-12: major components (>60%, based on MALDI-MS) are shown and the minor components are the higher oligomers 9–13, respectively; AO, aminooxy (AO)-functionalized 1,2-dihexadecyl-*sn*-glycero-3-phosphoethanolamine.

^c^DP, degree of polymerization of the major component.

In sum, the results presented here show the CLRs DC-SIGN, DC-SIGNR and MBP can bind to linear β1,2-gluco-oligosaccharides derived from *B. abortus* CβG with differing influence of chain length on the observable binding.

### Exploratory studies of the recognition of cyclic β1,2-glucan by DC-SIGN CLR

In additional experiments, we explored the recognition of intact forms of CβG by DC-SIGN-Fc (Supplementary Methods and Supplementary, Figure S2). His-Dectin-1 was included as a control protein. We analyzed the native form of *B. abortus* CβG with succinyl side chains and the NaOH-treated CβG with these side chains removed. As these CβGs are of relatively low-molecular weight (∼3 kDa, Figure 1) and cannot be readily retained on a nitrocellulose matrix, we explored the arraying of these together with other polysaccharides as positive and negative controls in the presence of a water soluble and photoactive terpolymer, sciPOLY3D, (Supplementary, Figure S2 and Supplementary, Table SI). The terpolymer consists of three components: poly(dimethylacrylamide) as the hydrophilic matrix, sodium salt of styrene sulfonate as the water solubility enhancer, and 4-methacryloxyl-oxy-benzophenone as the photo reactive group. This polymer allows immobilization of the molecules in a 3D matrix by UV irradiation forming covalent links between the capture molecules and the polymer and attachment of the polymer to the slide surface. As shown in Supplementary, Figure S2, DC-SIGN-Fc gave robust binding signals with α-mannan of *Saccharomyces cerevisiae*, which is well known to be recognized by this receptor (Cambi et al. 2008). Binding could be detected to the two CβG forms also to the β1,3-glucans NSG (neutral soluble β-glucan) and PGG (poly-(1,6)-d-glucopyranosyl-(1,3)-d-glucopyranose), as we have shown previously and in agreement with our observation that DC-SIGN bound to β1,3-gluco-oligosaccharides with DP 2 and 3 (Palma et al. 2006). Both NSG and PGG, as predicted, were bound by His-Dectin-1.

## Discussion

Glycan microarray technology has become established as a powerful means of glycan ligand discovery in studies of recognition systems in functional glycomics. There is an ever-demanding need to broaden the repertoire of sequence-defined oligosaccharide probes in order to facilitate the studies of glycan recognition in diverse biological systems. In the present study, we address the methodological challenge in obtaining β1,2-linked glucan oligosaccharides with differing chain lengths and their conversion into NGL probes for microarray construction to enable studies of their recognition by proteins. To achieve this, our strategy has been to partially depolymerize CβG of *B. abortus*, after removal of the succinyl side chains. Following detailed characterization of the oligosaccharides by mass spectrometry and NMR, the gluco-oligosaccharides were conjugated to the lipid reagent by oxime-ligation reaction using conditions we optimized for these hard-to-derivatize oligomers. NGL probes with differing chain lengths ranging from DP 2–13 were thus obtained.

The β1,2-linked CβGs are produced by different bacteria of the Proteobacteria phylum and occur mostly in the periplasm, but they can also be secreted as extracellular polysaccharides, to which important biological activities have been attributed (Bontemps-Gallo and Lacroix, 2015). For example, the periplasmic CβG of the pathogen *B. abortus* is essential for bacterial virulence, whereas the secreted CβG mediates interactions with mammalian hosts (Briones et al. 2001; Arellano-Reynoso et al. 2005) and modulation of the activities of immune cells (Martirosyan et al. 2012; Degos et al. 2015). Secreted CβGs have been shown also to be important for invasion of the bacterial phytopathogen *Xanthomonas campestris pv campestris* and suppression of systemic immune responses in plants (Rigano et al. 2007). Linear β1,2-glucans have also been identified in some bacteria of the Proteobacteria phylum, for example in the opportunistic pathogen *Pseudomonas aeruginosa*, in which they have been shown to be involved in biofilm formation (Lequette et al. 2007). Despite the wide occurrence and striking bioactivities attributed to these biomolecules limited information exists about proteins that mediate their recognition.

In the present work, we analyze two additional CLRs of the mammalian immune system for their recognition of β1,2-gluco-oligosaccharides, following on from our earlier finding that DC-SIGN binds to this type of oligosaccharides (Palma et al. 2015). We analyze a different DC-SIGN construct, its closely related endothelial cell receptor DC-SIGNR, and serum MBP, and show that these CLRs share the common feature of binding to β1,2-linked glucose oligosaccharides printed as NGL probes. It has been previously shown by microarray screening and structural analysis of the CRDs in complex with mammalian-type oligosaccharides that DC-SIGN and DC-SIGNR have distinct ligand-binding properties (Feinberg et al. 2001; Guo et al. 2004): both receptors bind high-mannose oligosaccharides; but DC-SIGN can additionally interact strongly with fucosylated Lewis^a^ and Lewis^x^-related oligosaccharides. Serum MBP binds to oligosaccharides bearing terminal fucose, mannose or GlcNAc with broad specificity (Drickamer and Taylor, 2015). The binding that we observe to the gluco-oligomers may reflect the mode of binding of these receptors to the shared high-mannose oligosaccharide ligands through the equatorial 3-hydroxyl and 4-hydroxyl groups (Drickamer and Taylor, 2015). This interpretation will need to be corroborated by solving the structures of the CRD-oligosaccharide complexes.

Our findings that DC-SIGN, DC-SIGNR and MBP can interact with β1,2-gluco-oligosaccharides derived from *B. abortus* CβG, raised the important question of whether the natural intact CβG forms are recognized and thereby involved in the triggering of these receptors of the immune system. Conformational differences between linear and cyclic β1,2-linked oligosaccharides have been described (Mimura et al. 1996). Our exploratory analyses suggest that DC-SIGN can interact with intact CβG forms. Further investigations of these interactions are required and of their involvement on the immuno biological effects observed with *B. abortu*s CβG and β1,2-linked glucans.

The availability of β1,2-linked glucose oligosaccharide probes derived from CβG and their effective presentation in microarrays will enable identification of oligosaccharide epitopes recognized on CβGs by other proteins. The β1,2-linked CβGs produced by bacteria exhibit structural diversity as they can occur in the unsubstituted form, or substituted at glucose C6 with anionic groups, such as succinyl (Roset et al. 2006) as in the case of the present work, phosphoglycerol (Miller et al. 1987), or methylmalonate (de Iannino and Ugalde, 1989). These substitutions as well as branching of the backbone chain with β1,6-linked glucose also occur in linear β1,2-glucans (Lequette et al. 2007). The strategies presented here could well be adapted to these various types of β1,2-linked glucans and may also be applied to the study of the influence of substitutions and branching on their recognition by proteins. The perfection of the sciPOLY3D polymer printing and development of new strategies to generate arrays of the intact CβGs will be important in the unraveling of these recognition systems.

## Materials and methods

### Oligosaccharides and recombinant proteins

A gluco-oligosaccharide fraction with DP 13 from curdlan (with β1,3-linkage), dextran-derived fractions (with α1,6-linkage) with DP 8, 9 and 10 and *Cyanobacterium* gluco-oligosaccharides (α1,2-linkage) with DP 2–9 as major components (Cyano-2 to Cyano-9) were prepared as described (Palma et al. 2015). Recombinant, tetrameric DC-SIGN and DC-SIGNR (complete extracellular domains, lacking the transmembrane domain) were made and purified as described previously (Mitchell et al. 2001). These were analyzed in the microarrays in a biotinylated form (DC-SIGN-bio and DC-SIGNR-bio, respectively), prepared as described previously (Carroll et al. 2010); MBP purified from human serum (Haurum et al. 1993; Jensenius 1995) was provided by Jens Christian Jensenius (Aarhus University, Denmark); murine Dectin-1 CRD with an N-terminal His6-tag (His-Dectin-1) was purchased from Sino Biologicals (Beijing, China). Solvents used are all of analytical grade and the compositions of the solvents are by volume throughout the study unless specified otherwise.

### Preparation of gluco-oligosaccharides from CβG

CβG, consisting of 13–23 glucose residues, was isolated from *B. abortus* essentially as described (Ciocchini et al. 2007) with some minor modifications. Cells from 200 mL of stationary phase cultures of *B. abortus* strain were grown for 48 h at 37°C (200 rpm) and harvested by centrifugation at 8000 ×*g* for 10 min at 4°C. Cell pellets were extracted with ethanol (70% ethanol, 1 h at 37°C). The ethanolic extracts were centrifuged, and the supernatants were concentrated and subjected to gel filtration on a Bio-Gel P6 column (1.8 × 78 cm). Columns were eluted at room temperature with 0.5% formic acid at a flow rate of 9 mL h^−1^, and 1.5 mL fractions were collected. Fractions corresponding to CβG were pooled, concentrated and lyophilized. CβG was initially treated with 0.1 M NaOH at 40°C for 60 min to remove the succinyl side chains. Following neutralization by addition of 3 M HCl to pH 7.0, the reaction mixture was desalted on a G10 column (1.6 × 30 cm). The side chain-removed CβG eluting at the void volume was collected and freeze-dried. The successful removal of the succinyl side chains was confirmed by matrix-assisted laser desorption/ionization mass spectrometry (MALDI-MS) analysis.

Small-scale experiments were performed initially to optimize the conditions for mild acid hydrolysis of the NaOH-treated CβG to obtain oligosaccharide fractions with DP 2–13. For this, 5 mg of the lyophilized CβG was dissolved in 500 µL of 0.01 M HCl in a V-shaped glass vial. The mixture was heated, with stirring, to 100°C in a heating block. For monitoring of the reaction progress, aliquots (50 µL) of the reaction solution were taken out at various reaction times (0, 30, 60, 120, 150, 180, and 210 min), cooled on ice and neutralized by addition of NaOH solution (0.1 M) before injection to an FPLC system equipped with a Superdex Peptide column (PC 3.2/30, GE Healthcare, Uppsala, Sweden). The column was eluted with deionized water at a flow rate of 18 mL h^−1^ and the eluent was monitored with a refractive index detector.

For large-scale preparation, the reaction time of 120 min was selected. Thus, 25 mg NaOH-treated CβG was dissolved in 2.5 mL HCl (0.01 M) and the mixture was incubated at 100°C for 120 min. The reaction was stopped by neutralization with NaOH (0.1 M) and the mixture was desalted on the Sephadex G10 column. The desalted hydrolysis products were fractionated on a Bio-Gel P4 column (1.5 × 100 cm) by elution with deionized water at a flow rate of 15 mL h^−1^. The elution was monitored on-line by refractive index and fractions were pooled according to their glucose units.

The pooled fractions were freeze-dried, and quantified by orcinol assay for glucose content (Chai et al. 2003). For high-performance silica gel TLC analysis, an aliquot (∼2 µg) of each fraction was applied to the aluminum-backed plate and a solvent system of *n*-propanol/water (8:3) was used for development. The gluco-oligosaccharide bands were detected by orcinol staining (Chai et al. 2003).

### Preparation of β1,2-gluco-oligosaccharide NGLs

The β1,2-linked gluco-oligosaccharides were converted into NGLs by oxime-ligation with the lipid reagent amino oxy-functionalized 1,2-dihexadecyl-*sn*-glycero-3-phosphoethanolamine (AOPE) (Liu et al. 2007). For β1,2-linked gluco-oligosaccharides with DP < 7, and β1,3-linked oligosaccharide with DP 13 (included as a standard control probe), the conjugation conditions were as described (Liu et al. 2007). In brief, 50 nmol of gluco-oligosaccharide in a glass vial were dried by lyophilization before addition of 100 nmol AOPE (in 20 µL of CHCl_3_/MeOH/H_2_O, 10:10:1). The solvent of the mixture was evaporated to dryness under an N_2_ stream. The content was re-dissolved in 50 µL of the same solvent and the mixture was incubated at ambient temperature (22°C) for 16 h before solvent evaporation in a heating block at 60^°^C for ∼1 h.

For β1,2-linked gluco-oligosaccharides with DP > 7, the reaction conditions were optimized to obtain higher conjugation yields. In exploratory studies, using dextran oligosaccharides DP 8, 9 and 10 as standards, the effects of reaction time and temperature were assessed. The reaction time was extended to 48 and 96 h and the reaction temperature was raised from ambient temperature to 50 or 80°C without any apparent improvement in reaction yield. The solvent in the reaction mixture was changed to acidic by addition of 2 µL of acetic acid or alkaline by addition of 2 µL of triethylamine. For further improvement of solubility of the higher oligomers of the gluco-oligosaccharides, DMSO was included in the reaction solvent. For comparison, two solvent systems CHCl_3_/MeOH/H_2_O (25:25:8) and CHCl_3_/MeOH/DMSO (25:25:8) were used in the following three procedures using 50 nmol of oligosaccharide and 1250 nmol of AOPE: (a) oligosaccharide and AOPE in 100 µL CHCl_3_/MeOH/H_2_O, (b) the oligosaccharide was dissolved in 15 µL DMSO before addition of 100 µL CHCl_3_/MeOH/H_2_O containing the required 1250 nmol of AOPE and (c) the procedure was identical to (b) apart from a solvent of CHCl_3_/MeOH/DMSO was used instead of CHCl_3_/MeOH/H_2_O. All the reactions were carried out at 80°C for 96 h. After reaction, the volatile solvent was evaporated under a stream of N_2_ and DMSO was removed by repeated co-evaporation with a small amount of water by lyophilization. Procedure (c) was selected for preparation of the higher oligomers (DP > 7) of β1,2-linked gluco-oligosaccharides.

NGLs of DP 2–5 were purified by semi-preparative HPTLC and those with DP 6–13 were purified using silica cartridge (Chai et al. 2003). Purified NGLs were analyzed by HPTLC using CHCl_3_/MeOH/H_2_O (60:35:8) as the development solvent and detected by primulin and orcinol staining (Chai et al. 2003).

### Analysis of the oligosaccharides and their NGLs

MALDI-MS in the positive-ion mode was carried out on a Tof Spec-2E instrument (Micromass, Manchester, UK) for analyses of the CβG polysaccharide, oligosaccharide fractions with DP 5–13 and all the NGLs. Sample solutions (1 µL, containing 1–10 pmol µL^−1^ in H_2_O for the poly- and oligosaccharides, and CHCl_3_/MeOH/DMSO, 25:25:8, for NGLs) were deposited on the sample target together with the matrix of 2-(4-hydroxyphenylazo) benzoic acid. Laser energy was 20% (coarse) and 60% (fine), and resolution was at 3000.

Negative-ion electrospray mass spectrometry (ESI-MS) was used for shorter oligosaccharides (DP 2–4). Collision-induced dissociation tandem mass spectrometry (ESI-CID-MS/MS) was used for sequence and linkage analysis for the heptasaccharide. ESI-MS and CID-MS/MS were carried out on a Q-TOF mass spectrometer (Micromass, Manchester, UK). Nitrogen was used as desolvation and nebulizer gas at a flow rate of 250 and 150 L h^−1^, respectively. Source temperature was 80°C, and the desolvation temperature was 150°C. A cone voltage of 50 V was used and the capillary voltage was maintained at 3 kV. MS/MS product-ion spectrum was obtained from CID using Argon was used as the collision gas at a pressure of 0.17 MPa for the CID-MS/MS experiment. The collision energy was at 17 V. For analysis, oligosaccharides were dissolved in acetonitrile/water (1:1), typically at a concentration of 15 pmol µL^−1^, of which 5 µL was loop injected. Solvent (acetonitrile/2 mM ammonium bicarbonate, 1:1) was delivered by a Harvard syringe pump (Harvard Apparatus, Holliston, MA) at a flow rate of 10 µL min^−1^.

For NMR analysis, the CβG-derived fraction with DP 7 (150 µg) was co-evaporated with ^2^H_2_O (99.9 atom% ^2^H_2_) twice by lyophilization and dissolved in 550 µL of high-quality ^2^H_2_O (100.0 atom% ^2^H_2_), containing 0.1 µL of acetone. ^1^H NMR spectrum was acquired on Varian (Palo Alto, CA) Unity-600 (599.89 MHz ^1^H) spectrometer at 25°C and processed with standard Varian software. The observed ^1^H chemical shifts were relative to internal acetone (2.225 ppm).

### Carbohydrate microarray analyses

For preparation of the microarray, the gluco-oligosaccharide NGL probes (Table I) were printed onto 16-pad nitrocellulose-coated glass slides in duplicate at two levels, 2 and 5 fmol spot^−1^, as described (Palma et al. 2015).

Microarray-binding analyses, performed using AlexaFluor-647-labeled Streptavidin as final readout of protein binding, imaging and data analysis were carried out essentially as described (Liu et al. 2012). The biotinylated DC-SIGN and DC-SIGNR extracellular domains were analyzed at 50 µg mL^−1^, diluted in 0.02% casein (Pierce blocking solution) in HBS (5 mM HEPES buffer, pH 7.4, 150 mM NaCl) with addition of 1% BSA and 5 mM CaCl_2_ (Ca-Casein/BSA); MBP was analyzed at 4 µg mL^−1^ in the blocking solution Ca-Casein/BSA, followed by a biotinylated rabbit anti-MBP (Haurum et al. 1993) diluted at 3 µg mL^−1^ in the same blocker; His-Dectin-1 was analyzed pre-complexed with mouse monoclonal anti-poly-histidine and biotinylated anti-mouse IgG antibodies, both from Sigma, at a ratio of 1:3:3 (by weight) as described (Palma et al. 2015), and diluted to the final concentration of 20 µg mL in the blocking solution 3% (w/v) BSA from Sigma (A8577) in HBS.

## Supplementary data

Supplementary data for this article are available online at http://glycob.oxfordjournals.org/.

## Funding

This work was supported by the Wellcome Trust grants WT093378MA and WT099197MA to T.F. and W.C., and the UK Research Councils’ Basic Technology Initiative “Glycoarrays” (GRS/79268) and EPSRC Translational Grant (EP/G037604/1) to T.F.; Natural Science Foundation of China (31201384) to H.Z.; and the Fundação para a Ciência e Tecnologia (FCT): FCT Investigator and PTDC/QUI-QUI/112537/2009 to A.S.P., RECI/BBB-BEP/0124/2012 and UID/Multi/04378/2013 grants. H.Z. was supported by China Scholarship Council (CSC: 2008679005). Funding to pay the Open Access publication charges for this article was provided by Wellcome Trust Grant WT099197MA.

## Conflict of interest statement

None declared.

## Abbreviations

CLRs, C-type lectin receptors; CRDs, carbohydrate recognition domains; CβG, cyclic β1,2-glucan; DC-SIGN, dendritic cell-specific ICAM-3-grabbing nonintegrin; DP, degrees of polymerization; MBP, mannose-binding protein; MS, mass spectrometric; NGL, neoglycolipid.

## Supplementary Material

Supplementary Data
