# Supplementary material for: Generation and characterization of β1,2-gluco-oligosaccharide probes from Brucella abortus cyclic β-glucan and their recognition by C-type lectins of the immune system
Source: Glycobiology. 2016 Oct 18;26(10):1086–96. doi: 10.1093/glycob/cww041 (PMC5072146; doi:10.1093/glycob/cww041)
Supplement: Supplementary Data [file supp_26_10_1086__index.html]

Generation and characterization of β1,2-gluco-oligosaccharide probes from Brucella abortus cyclic β-glucan and their recognition by C-type lectins of the immune system — Generation and characterization of β1,2-gluco-oligosaccharide probes from Brucella abortus cyclic β-glucan and their recognition by C-type lectins of the immune system — Generation and characterization of β1,2-gluco-oligosaccharide probes from Brucella abortus cyclic β-glucan and their recognition by C-type lectins of the immune system — Supplementary Data 

# Generation and characterization of β1,2-gluco-oligosaccharide probes from *Brucella abortus* cyclic β-glucan and their recognition by C-type lectins of the immune system

## Supplementary Data

Supplementary Data

- Supplementary Data - Pdf file
