# Supplementary material for: Generation and characterization of β1,2-gluco-oligosaccharide probes from Brucella abortus cyclic β-glucan and their recognition by C-type lectins of the immune system
Source: Glycobiology. 2016 Oct 18;26(10):1086–96. doi: 10.1093/glycob/cww041 (PMC5072146; doi:10.1093/glycob/cww041)
Supplement: Supplementary Data [file supp_cww041_cww041supp.pdf]

Generation and characterization of β1,2-gluco-oligosaccharide probes from *Brucella abortus* cyclic β-glucan and their recognition by C-type lectins of the immune system

Hongtao Zhang[1,2], Angelina S. Palma[1,3], Yibing Zhang[1], Robert A Childs[1], Yan Liu[1], Daniel A. Mitchell[4], Leticia S. Guidolin[6], Wilfried Weigel[5], Barbara Mulloy[1], Andrés E. Ciocchini[6], Ten Feizi[1] and Wengang Chai[1]

[1]Glycosciences Laboratory, Department of Medicine, Imperial College London, London W12 0NN, UK; [2]Key Laboratory of Carbohydrate Chemistry and Biotechnology, Ministry of Education, School of Biotechnology, Wuxi 214122, Jiangnan University, China; [3]UCIBIO-REQUIMTE, Department of Chemistry, Faculty of Science and Technology, NOVA Universidade de Lisboa, 2829-516 Caparica, Portugal; [4]CSRI-UHCW, Walsgrave Campus, University of Warwick, Coventry, CV2 2DX, UK; [5]Instituto de Investigaciones Biotecnológicas “Dr. Rodolfo A. Ugalde”, Instituto Tecnológico de Chascomús (IIB-INTECH), Universidad Nacional de San Martín, San Martín 1650, Buenos Aires, Argentina; [6]SCIENION AG, Volmerstrasse 7b, 12489 Berlin, Germany.

Supplementary Data

Supplementary Methods

Microarray analyses to determine if DC-SIGN can bind the native and NaOH-treated CβG in a polysaccharide array

A new method of microarray construction was used to evaluate whether the native CβG and NaOH-treated CβG with the succinyl side chains removed (CβG-NaOH) can be bound by DC-SIGN. The method developed by Scienion, involves the arraying of macromolecules in the presence of 300 mM sodium phosphate buffer (pH 7.5) containing a water soluble polymer, sciPOLY3D, at 1 mg of per ml. After printing, the slides are exposed to UV light to induce covalent attachment of the polysaccharide to the 3D polymer matrix and immobilization of the co-polymer formed to the slide surface. We analysed Dectin-1 binding as a control protein, and show data including five additional polysaccharides (Figure S2 and Table S1). The polysaccharides were arrayed at the concentrations indicated in Table S1. Cyanine 3 (20 ng/ml) was

included as a tracer in the printing solution for spot location. Binding analyses were performed with DC-SIGN CRD fused to human IgG at the C-terminus (DC-SIGN-Fc) and murine Dectin-1 CRD with an *N*-terminal His6-tag (His-Dectin-1), both purchased from Sino Biologicals (Beijing, China). After re-hydrating the arrayed slides with HBS (5 mM HEPES buffer pH7.4, 150 mM NaCl) including 0.05% (v/v) Tween 20 (designated washing buffer) they were overlaid with the protein solutions. DC-SIGN-Fc was overlaid at 10 or 100 µg/ml diluted in 1% BSA in HBS with addition of 0.05% v/v Tween 20 and 5 mM $CaCl_2$ (diluent). Bound DC-SIGN-Fc was detected with biotinylated goat anti-human-IgG (Vector) at 10 µg/ml in diluent. His-Dectin-1 was analysed pre-complexed with mouse monoclonal anti-poly-histidine and biotinylated anti-mouse IgG antibodies, both from Sigma, at a ratio of 1:3:3 (by weight), and diluted in diluent to a final His-Dectin-1 concentration of 30 µg/ml. Binding was detected with AlexaFluor-647 and data analysis was carried out essentially as described (Liu et al., 2012).

Supplementary Figures

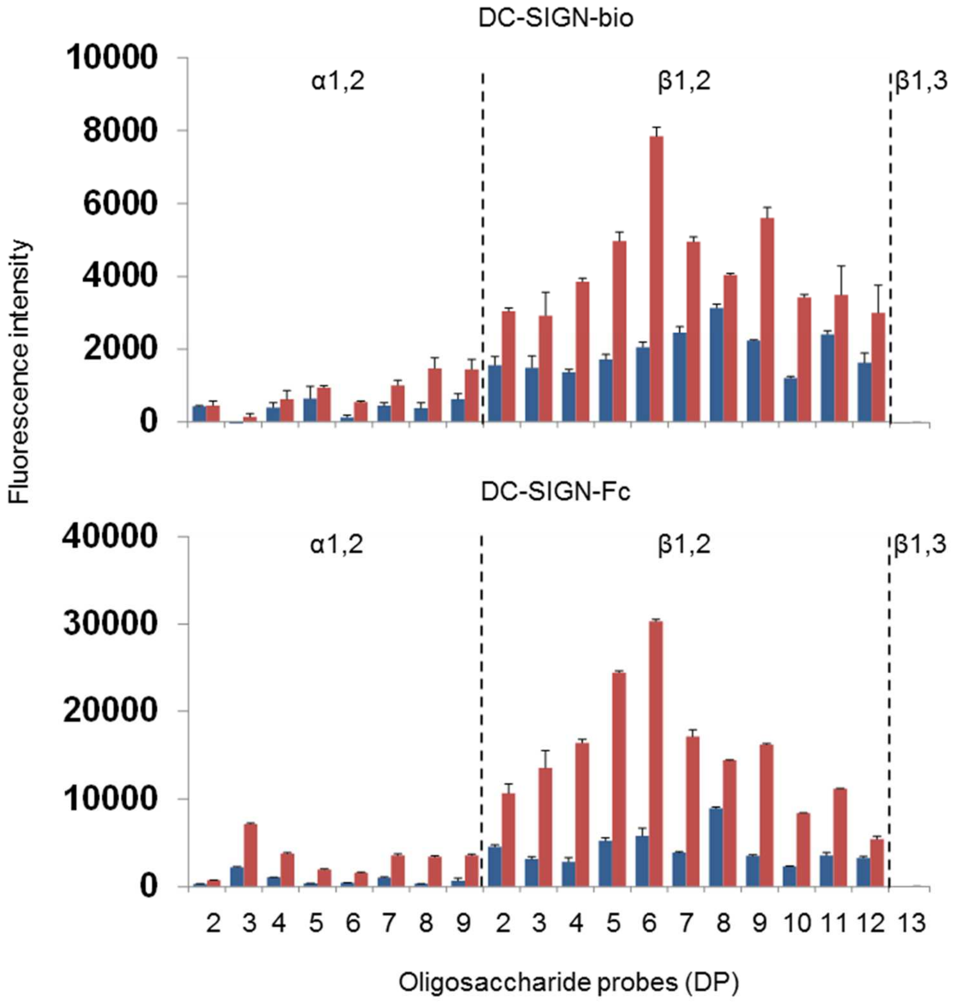

Figure S1: Comparison of binding signals obtained with biotinylated tetrameric DC-SIGN-bio (50 µg/ml) and homodimeric DC-SIGN-Fc (2 µg/ml) published in (Palma et al., 2015).

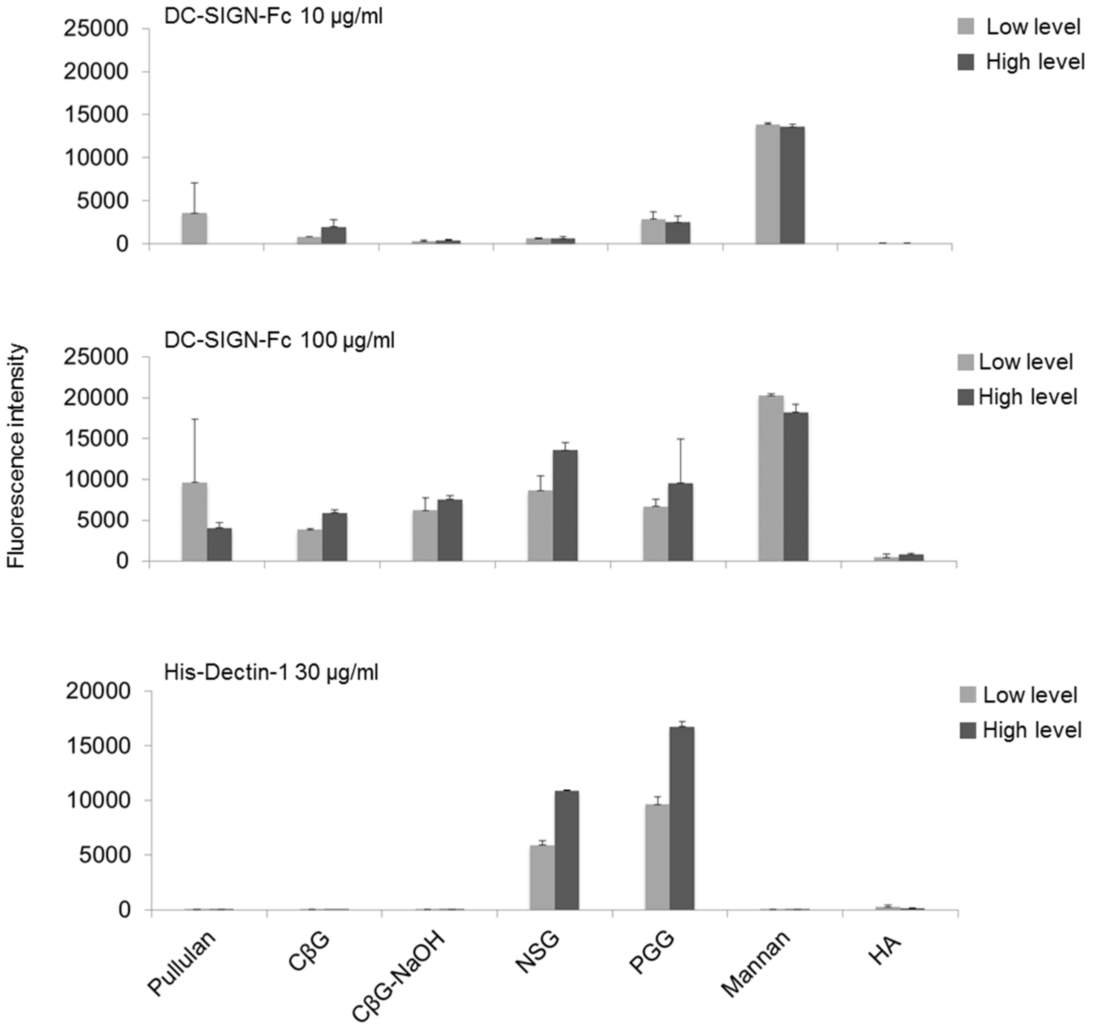

Figure S2: Microarray analyses of the binding of DC-SIGN-Fc and His-Dectin-1 to polysaccharides arrayed in the presence of a sciPOLY3D polymer. Each polysaccharide was printed at two different concentrations as indicated in Table S1 and depicted as low and high level in the figure.

Supplementary Table 1. List of polysaccharide samples arrayed at the concentrations indicated.

Polysaccharide[a]	Predominant oligosaccharide sequence	Mean molecular mass (kDa)[b]	Reference	Concentration (mg/ml)
1. Pullulan	α1,4- and α1,6-Glc	45-250	(McCleary and Matheson, 1987)	0.1<br>0.5
2. CβG	Cyclic β1,2-Glc with succinyl side chains	2.6-3.7[b]	(Ciocchini et al., 2007) and this study	0.1<br>0.5
3. CβG-NaOH	Cyclic β1,2-Glc	2.6-3.7[b]	This study	0.1<br>0.5
4. NSG	β1,3-Glc with β1,6-monoglucosyl branches	10-20	(Hong et al., 2003)	0.1<br>0.5
5. PGG	β1,3-Glc with β1,6-monoglucosyl branches	120-205	(Jamas et al., 1991)	0.1<br>0.5
6. Mannan	α1,6-Man	37	(Cambi et al., 2008)	0.1<br>0.5
7. HA	-3GlcAβ1,4GlcNAcβ1-	>400	(Chai et al., 2001)	0.3<br>1

[a] Pullulan, from *Pullularia pullulans* (Megazyme); CβG, Cyclic β-glucan from *Brucella abortus*; CβG-NaOH, CβG treated with alkali to remove the succinyl side chains; NSG, neutral soluble β-glucan, from *Saccharomyces cerevisiae* (Biothera); PGG, abbreviation of poly-(1,6)-D-glucopyranosyl-(1,3)-D-glucopyranose, from *S. cerevisiae* (Biothera); Mannan, from *S. cerevisiae* (Sigma); HA, from bovine vitreous humor (Sigma).

[b] MALDI-MS analysis carried out on CβG indicated a degree of polymerization (DP) 16-23 with DP 17 as a major component (Figure 1).

Supplementary References

Cambi A, Netea MG, Mora-Montes HM, Gow NA, Hato SV, Low man DW, Kullberg BJ, Torensma R, Williams DL, Figdor CG. 2008. Dendritic cell interaction with Candida albicans critically depends on N-linked mannan. *J. Biol. Chem*. 283: 20590-20599.

Chai W, Beeson JG, Kogelberg H, Brown GV, Lawson AM. 2001. Inhibition of adhesion of Plasmodium falciparum-infected erythrocytes by structurally defined hyaluronic acid dodecasaccharides. *Infect. Immun. 69: 420-425.*

Ciocchini AE, Guidolin LS, Casabuono AC, Couto AS, de Iannino NI, Ugalde RA. 2007. A glycosyltransferase with a length-controlling activity as a mechanism to regulate the size of polysaccharides. *Proc. Natl. Acad. Sci. U. S. A.* 104: 16492-16497.

Hong F, Hansen RD, Yan J, Allendorf DJ, Baran JT, Ostroff GR, Ross GD. 2003. β-glucan functions as an adjuvant for monoclonal antibody immunotherapy by recruiting tumoricidal granulocytes as killer cells. *Cancer Res.* 63: 9023-9031.

Jamas S, Easson DDJ, Ostroff GR, Onderdonk AB. 1991. Glucans a novel class of macrophage activating immunomodulators. *ACS Symp. Ser.* 469: 44-51.

Liu Y, Childs RA, Palma AS, Campanero-Rhodes MA, Stoll MS, Chai W, Feizi T. 2012. Neoglycolipid-Based Oligosaccharide Microarray System: Preparation of NGLs and Their Noncovalent Immobilization on Nitrocellulose-Coated Glass Slides for Microarray Analyses. Methods Mol. Biol. *808*: 117-136.

McClearyBV, Matheson NK. 1987. Enzymic analysis of polysaccharide structure. Adv. Carbohyd.Chem. Bi. *44*: 147-276.

Palma AS, Liu Y, Zhang H, Zhang Y, McCleary BV, Yu G, Huang Q, Guidolin LS, Ciocchini AE, Torosantucci A, Wang D, Carvalho AL, Fontes CM, Mulloy B, Childs RA, Feizi T, Chai W. 2015. Unravelling glucan recognition systems by glycome microarrays using the designer approach and mass spectrometry. Mol. Cell Proteomics. *14*: 974-988.
